# Novel behavioral tasks for the measurement of social motivation in mice: a comparison across strains

**DOI:** 10.3389/fnbeh.2025.1678147

**Published:** 2026-01-06

**Authors:** Caitlyn Wells, Kendall P. Huddleston, Steven Brown, Fatima Razzaq, Donald Chick, Tiffany D. Rogers

**Affiliations:** Department Psychology, Middle Tennessee State University, Murfreesboro, TN, United States

**Keywords:** autism, BTBR, sociability, social approach, social motivation, social behavior, mouse models

## Abstract

**Introduction:**

Social isolation, reduced social interaction, and social anhedonia are associated with a range of neuropsychiatric conditions. While the search for novel pharmacological agents to treat social symptoms persists, more precise social behavior measures in pre-clinical animal models are needed to make the most accurate predictions of therapeutic outcomes.

**Methods:**

In the current study, we propose two novel behavioral tasks to measure social motivation in mouse models. We define social motivation as the willingness to exert effort to access a social partner. The first social motivation test, the weighted door task, requires a mouse to push open a one-way, weighted door that increases in weight across successive trials to access a social partner behind the door. The second social motivation test, the ladder task, requires a mouse to climb a ladder that increases in steepness across trials to access a social partner on a platform at the top of the ladder. To validate these tasks, we compared behavioral outcomes across three common inbred strains, C57BL/6J, DBA/2J, and BTBR T + Itpr3 tf /J. Social motivation outcomes were then compared to outcomes in two standard social behavior tests: the three-chamber task and the free dyadic social interaction task.

**Results:**

Following behavioral testing, we found that each strain displayed distinct behavioral responses in social motivation tasks with BTBR mice demonstrating low social motivation, DBA mice demonstrating high social motivation, and C57 mice demonstrating conditionally high social motivation during low effort trials.

**Discussion:**

When combined with standard social behavior testing, our measures provide more detailed social behavior phenotypes unique to each strain. In addition to allowing the creation of more complete social behavior ethograms, these tasks offer advantages as compared to existing conditioning-based behavior tasks measuring social motivation and reward such as the social conditioned place preference task and operant conditioning for social reward. The weighted door and ladder tasks leverage innate exploration behaviors that do not require prior learning which allows for more models, including those with memory, attention, and learning deficits, to be used. These pre-clinical measures of social motivation may prove useful in improving predictions of social behavior outcomes of proposed pharmacological interventions for clinical populations.

## 1 Introduction

Differences in social behavior are associated with a wide variety of neuropsychiatric and neurodevelopmental conditions. One of the core symptoms of autism spectrum disorder (ASD) is reduced or irregular social interaction (Lord et al., 2018). Likewise, reduced social interaction is a hallmark of social anxiety disorder (SAD) due to an overwhelming fear of social judgement or embarrassment (Zhang et al., 2023). Major depressive disorder (MDD) and schizophrenia-spectrum disorders (SSDs) are often associated with social anhedonia leading to reduced social communication, cooperation, and connectedness (Blanchard et al., 2001; Frick et al., 2021; Hanssen et al., 2018; Ritsner et al., 2018; Shovestul et al., 2022). In addition to differences in social behavior, individuals diagnosed with psychiatric and neurodevelopmental disorders more frequently report subjective feelings of loneliness and social disconnection as compared to nonclinical populations (Caple et al., 2023). Rates of loneliness in these clinical populations are correlated with increased risk of additional diagnoses. For example, individuals with neurodevelopmental disorders and high self-reported levels of loneliness are more likely to also be diagnosed with depression (Kwan et al., 2020). Given the prevalence and impact of reduced social interaction and perceived social isolation in clinical populations, it is increasingly important to reliably measure and treat these states.

Ongoing efforts to identify pharmacological treatments for social symptoms have identified promising options currently being evaluated in clinical trials (Bershad, 2023a,b; Fava et al., 2020; Lee et al., 2023; Parker et al., 2017). While these important advances provide pathways toward pharmacological intervention, multiple challenges still exist, including the heterogeneity of social behavior measurement and outcomes (Bartz et al., 2011; Kirkpatrick et al., 2014; Parker et al., 2017; Studerus et al., 2021). The inherently complex nature of social interactions and behaviors leads to the utilization of a wide array of heterogeneous behavioral measures and variable behavioral outcomes that are dependent on the measurement strategy, individual differences, and context.

One tactic to enhance our ability to measure and predict social behavior in clinical and preclinical research is to clearly define constructs that represent the full range of social behavior categories and design measurements for each of these specified categories. One potentially useful approach, as outlined in the Berridge-Robinson framework for addiction, would be to delineate the hedonic experience of a reward, the learning required to reliably access a reward, and the motivation to continue pursuing a reward (Berridge et al., 2009; Berridge and Robinson, 2016; Robinson et al., 2015). Likewise, social behavior may be subdivided into hedonic social reward, cognitive social learning, and effort-based social motivation. Recent brain imaging research has demonstrated overlapping neural circuits associated with social reward, social cognition, and social motivation. Social reward can include social approval, avoidance of social rejection, positive social touch, or the presentation of positive facial cues. While brain region activation is somewhat dependent on the reward type, classic reward-processing areas, such as the ventromedial striatum including the nucleus accumbens, are commonly associated with processing social reward (Kohls et al., 2013; Sherman et al., 2016; for review see Báez-Mendoza and Schultz, 2013 and Bhanji and Delgado, 2014). Social cognition includes learning processes such as decision making, social behavior prediction, observational learning, salience of social stimuli, and the ability to infer mental states of others. Social cognition tasks commonly elicit activation in the medial prefrontal cortex and the temporoparietal junction (Bitsch et al., 2018; Mars et al., 2012; Mitchell et al., 2005a,b; for review see Van Overwalle, 2009). Social motivation is the willingness to engage in social interaction or to exert effort for a social reward for self or other. Activation of brain regions including ventromedial prefrontal cortex and the dorsal anterior cingulate cortex are associated with making choices to exert effort for a social reward or to enact prosocial behavior (Forbes et al., 2024; Lockwood et al., 2024). Delineating social reward, social cognition, and social motivation creates clearly defined categories of social behavior for which specific and discrete behavioral measures can be developed to allow novel understandings of the neurobiological correlates of complex social behavior in animals and humans.

To this end, the current study proposes two novel behavioral measures for use in mice to quantify social motivation as a separate construct from social reward and social cognition. While many measures of social behavior tasks for use in mice currently exist, the majority measure either social reward (whether a mouse finds social interaction to be preferable or hedonically rewarding) or social cognition (whether a mouse can successfully utilize social memory and learning to attend to, identify, pursue, or avoid a social partner). A brief review of most common social behavior tasks currently used with mice highlights the need for tasks requiring the mouse to exert effort to access a social partner (e.g., —social motivation).

### 1.1 The three-chamber task

One widely used task utilized to quantify social behavior is the three-chamber task (Moy et al., 2009). This task is commonly divided into three phases in which a mouse is placed in a center chamber connected by open doorways to two side chambers. In the first phase the side chambers remain empty while the mouse is allowed to freely explore the arena and habituate. In the second phase a social stimulus, usually a same-sex conspecific, is placed in one of the side chambers and the subject mouse is allowed to freely move between the chambers. Experimenters can use this task to infer the valence of a social stimulus by providing an opportunity for the subject mouse to approach and investigate a social stimulus with positive valence or to display social avoidance for a social stimulus with negative valence. In the third phase, the same task can be used to test social interaction time with a familiar versus novel social stimulus. The second phase allows for measures of social reward by measuring time in sniffing the social stimulus or remaining in the same chamber as the social stimulus. The third phase can test for social cognition, as social memory is required to distinguish between familiar or novel partners. The third phase can also measure for preference of social novelty, or the social reward specific to an unfamiliar partner. However, as no effort other than approach to a non-moving social partner is required, this test does not allow for measurement of social motivation.

### 1.2 Free dyadic social interaction task

During the free dyadic social interaction task (FDSI) (Kraeuter et al., 2019), two mice are simultaneously released from opposite corners in an open field chamber and allowed to freely interact. The experimental mouse can explore a social partner in a more natural environment. The interaction is also dyadic such that the behaviors of both the experimental and partner mouse can be measured simultaneously. By measuring time spent in social approach, social avoidance, body contact, and distance between animals, social reward can be measured. The amount of time spent pursuing a moving social partner could also be interpreted as social motivation as some effort is required. However, it is not possible to control the movements of the partner mouse to consistently measure this type of effort driven behavior.

### 1.3 T-maze

During the T-maze task (Trezza et al., 2011), the subject mouse is placed at the base of a T-shaped maze with social stimuli placed in the two upper arms of the maze. As the subject mouse approaches the split in the T-shaped maze, it can choose to explore one of two social stimulus choices. The social stimuli can vary to compare preference for a dominant or submissive partner, a male or female stimulus, and so on. The positive or negative valence of a social stimulus can then be quantified by the amount of time spent in social approach and social investigation or by the amount of time spent in social avoidance. Social cognition and social memory can also be tested by providing social stimulus choices with which the subject mouse has prior experience. However, without the augmentation of the test with a type of barrier or physical obstacle, the T-maze does not allow for measurement of social motivation.

### 1.4 Social interaction conditioned place preference

During the social interaction conditioned place preference (siCPP) task (Panksepp and Lahvis, 2007), classical conditioning is used to pair environmental cues or contexts to social reward (being in the presence of other mice) or social isolation. The subject mouse is re-exposed to the environmental contexts after conditioning has taken place to allow the subject mouse to choose between the environments, which is assumed to indicate the mouse’s preference for the social reward or social isolation condition (Harda et al., 2022; Misiołek et al., 2023; Panksepp and Lahvis, 2007). The siCPP can be used to measure social reward and, more indirectly, social cognition as the task is dependent upon social memory and learning.

### 1.5 Operant conditioning

In addition to classical conditioning, operant conditioning can be utilized to infer social reward with mouse models. One commonly used operant conditioning paradigm allows the subject mouse to lever press to access a social stimulus. The social self-administration chamber (Med Associates), for example, allows a mouse to open a guillotine style door via lever press (Lee et al., 2025; Navarrete et al., 2024; Ramsey et al., 2023). This same chamber allows for a choice paradigm to offer either a social reward (social interaction) or an alternative such as the administration of a drug reward. In this paradigm, the task measures the relative reward value of a social partner as compared to the alternative reward. This task can also measure social motivation as lever pressing requires exerted effort from the experimental mouse to receive the opportunity for social interaction, and required effort can be increased across trial by using reinforced progressive ratio testing (Navarrete et al., 2024). While this task may be used to measure 4 four weeks as described by Ramsey et al. (2023), and conditioning that is dependent on the cognitive abilities of a given mouse model.

To fill the current need to measure social motivation in addition to the many behavioral measures of social reward and social cognition, the current study proposes two novel measurements of social motivation in mice to allow the examination of effort-based social motivation to access a social partner: (1) the weighted door task and (2) the ladder task. Both measures capitalize on innate exploratory behaviors and do not require conditioning prior to behavioral testing allowing use in a wider variety of mouse models including models of ASD that might have learning or attention deficits. As a modification of the weighted door task published by Borland et al. (2017), we created a novel measurement of social motivation in mice requiring mice to push open a weighted door to access a social partner. The task allows for explicit quantification of social motivation as the weighted door increases in weight across successive trials. Similarly, we developed the ladder task in which a subject mouse is placed at the base of a ladder and required to climb the ladder, which is placed at a progressively steeper angle across trials, to reach a social stimulus.

The current study aims to demonstrate the feasibility of using the weighted door and ladder tasks to quantify social motivation in mice by comparing social motivation across three inbred strains and across sex. Inbred strains were selected to represent a variety of social behavior phenotypes. C57 mice are generally characterized as having a high sociability and relatively low levels of anxiety-like behaviors (Eltokhi et al., 2020; Laarakker et al., 2011; Moy et al., 2007; Yang et al., 2012). DBA mice are generally characterized as having moderate or variable sociability with high rates of anxiety-like behaviors (Eltokhi et al., 2020; Ma et al., 2015; Moy et al., 2007; Zhang et al., 2012). BTBR mice represent an inbred strain commonly used as a mouse model of autism due to their low level of sociability and other concomitant behaviors such as deficits in attention and learning (Khan et al., 2025; McFarlane et al., 2008; Moy et al., 2007; Yang et al., 2012). A panel of health, reflex, olfactory, and motor tests were performed on each subject mouse to examine any potential confounding factors that may influence the interpretation of the weighted door and ladder task. And finally, commonly used social behavior tests were performed to allow for the comparison of social motivation and social reward outcomes.

## 2 Materials and methods

### 2.1 Animals

Adult male and female mice from three inbred strains, C57BL/6J, DBA/2J, and BTBR *T^+^ Itpr3^tf^*/J, were purchased from the Jackson Laboratory (Strains #000664, #000671, and #002282, respectively). A total of 60 mice (20 mice per strain; 30 mice per sex) were housed in sex-matched groups of five with food and water available ad libitum. The colony and experimental rooms were kept on a 12/12-h light/dark cycle and controlled for temperature and humidity. Mice were habituated to the colony room for at least 2 weeks prior to initiating experiments and were habituated to caregiver handling prior to behavioral tests. All mice were approximately 10 weeks old at the initiation of behavioral tests. All procedures conducted were approved by the Institutional Animal Care and Use Committee of Middle Tennessee State University and conducted in accordance with the National Institutes of Health (NIH) Guidelines for the Care and Use of Laboratory Animals.

### 2.2 Testing procedures

During the first week post-arrival at the animal facilities, mice were examined for coat health and tagged for identification with numbered ear tags (Product #24221; Fine Science Tools). The following week, mice were tested for typical home cage behavior, reflexes, olfactory function, and motor coordination. Upon the completion of these measurements and the 2 week habituation period, mice were weighed, and behavior measurements were initiated. Mice were first tested for anxiety on the elevated plus maze (EPM), then for sociability in the three-chamber task and free dyadic social interaction (FDSI) task, and lastly for social motivation in the weighted door and ladder task. For all behavioral tasks using stimulus mice (FDSI, Three-Chamber, Weighted Door, and Ladder Task), experimental and stimulus mice were habituated to the arenas and pencil cups, respectively, prior to testing. Habituation to the arenas was performed by allowing the experimental mouse 10 min to explore the arena prior to the presentation of the stimulus mouse. Habituation to the pencil cups was performed by placing each stimulus mouse in the inverted pencil cup for 10 min, three times per day for two consecutive days prior to the initiation of behavior trials. A total of 20 stimulus mice (10 males and 10 females) were used as matched conspecifics to experimental mice. All stimulus and experimental mice were naïve prior to behavioral measures, and the behavior of the stimulus mice was not measured or included in the behavioral data of the experimental mice. Prior to each behavioral measurement, experimental and stimulus mice were habituated in the behavioral testing room for at least 1 h. Estrous cycles were not measured for female mice to prevent sex-based confounds associated with stress and increased handling during vaginal lavage. Each of the behavioral tasks, including the social and nonsocial conditions of the ladder task described below, were separated by a 24-h period. All behavioral arenas were cleaned with 70% ethanol and allowed to dry between trials and again between each experimental mouse. All behavioral tasks were conducted under fluorescent laboratory lighting during the light hours of the vivarium’s light/dark cycle.

### 2.3 Home cage behaviors

Prior to behavioral tests, mice were observed for typical home cage behaviors. Nest building was evaluated by placing a new cotton nestlet (Ancare) in each cage at approximately 8:00a. Observations of nest-building were then taken at approximately 12:00p and 4:00p and just before the lights off cycle began at 7:00p. Observations were made for a total of 20 min per time point throughout the day, resulting in a total of 1 hour of observation per cage. The percentage of cages exhibiting typical nest building (scoring either a 4 or 5 on the rating scale published by Deacon) were recorded during the last observation of the day (Deacon, 2006). During these same observations, mice were observed for sleeping in huddled groups rather than solitary sleeping positions. Mice were also monitored during observations for any aberrant behaviors.

### 2.4 Neurological reflexes

One week prior to behavioral testing, mice were tested for typical neurological reflexes. Vibrissae orienting, grasping, visual placing, and acoustic startle reflexes were examined (Crawley, 1999). Vibrissae orienting was tested by touching a cotton swab to the whiskers and observing whether the mouse’s head oriented toward the cotton swab. Grasping reflex was measured by observing the mouse’s ability to grasp a metal grid with the forepaws and hind paws. Visual placing reflex was measured by suspending each mouse by the base of the tail just above a metal grid and observing the extension of the forelimbs and raising of the head as the mouse is lowered. Acoustic startle reflex was measured by observing a startle response following a loud sound. Expression of reflexes was quantified as either present or absent and recorded as a percentage of mice per strain.

### 2.5 Olfactory test

Olfaction was evaluated by the buried food test (Machado et al., 2018; Moy et al., 2004). Mice were exposed to a novel food, Kellogg’s Fruit Loops Cereal®, 2–3 days prior to the test to prevent neophobia. At the initiation of testing, mice were placed in a new, clean home-cage filled with 3 cm of bedding. Mice were allowed to habituate to the new cage for 5 min and then removed. The novel food was buried 1 cm underneath the bedding. Mice were then returned to the cage and given 15 min to uncover the buried food. Latency to uncover the buried food was measured in seconds.

### 2.6 Rotarod test

Motor function and coordination was evaluated by the rotarod test (Deacon, 2013; Med Associates). Mice were placed on an accelerating, rotating rod that steadily increased in speed from 4–30 RPMs over the course of 5 min per trial. Each mouse was tested in three consecutive five-minute trials. Each trial ended when the subject mouse either fell from the rotarod or completed one full passive rotation by holding onto the rod as it spun. Latency to fall or perform a passive rotation was recorded in seconds by Med-PC software.

### 2.7 Elevated plus maze

Anxiety-like behaviors were tested by the EPM (Walf and Frye, 2013). The EPM arena consists of two arms enclosed by walls and two open arms without any surrounding barrier. Each arm of the EPM was 10 cm wide and 44 cm long, and the arena was elevated approximately 60 cm from the ground. Increased exploration of open arms indicates lower anxiety-like behaviors. Each mouse underwent one five-minute trial which began when the mouse was placed in the center of the maze. Each trial was video recorded, and time spent in the open arms and overall distance travelled was measured in seconds by Noldus Ethovision (Version 15) software.

### 2.8 Free dyadic social interaction task

Social approach and avoidance behaviors were evaluated by the FDSI test (Kraeuter et al., 2019). The FDSI was performed in a 30 cm by 30 cm opaque acrylic open field arena with 35 cm high walls. Both the subject mouse and a partner mouse were placed on opposing corners of the arena, and the trial began when the mice were simultaneously released. Partner mice were unfamiliar to the subject mouse and were strain-, age-, sex-, and weight-matched to the subject mouse. Each subject mouse was tested in a single five-minute trial, and social interactions between the two mice were video recorded from above. Noldus Ethovision (Version 15) software was then used to determine nose to nose sniffing, nose to tail sniffing, and body contact. Social approach was hand recorded by researchers blinded to strain and sex and was defined as walking movements of the subject mouse toward the partner mouse in which the subject mouse’s nose was oriented toward the partner mouse. Social avoidance was also hand recorded by a blinded researcher and was defined as walking movements of the subject mouse away from the partner mouse in which the subject mouse’s nose was oriented away from the partner mouse. All measures were recorded in seconds.

### 2.9 Three-chamber task

The three-chamber task was used to assess general sociability and preference for social novelty (Moy et al., 2009). The three-chamber arena, a 60 cm by 30 cm opaque acrylic box, consisted of one center chamber and two side chambers, each accessible by an opening. Inverted empty pencil cups were placed in each side chamber. The task consisted of three ten-minute phases. During the first phase, mice habituated to the arena while freely exploring. During the second phase, one strain-, age-, sex-, and weight-matched stimulus mouse was placed inside one of the inverted pencil cups while the other pencil cup remained empty. During the third phase, the first stimulus mouse, now the familiar social stimulus, remained on one side of the arena, while a second matched stimulus mouse, the novel social stimulus, was placed inside a pencil cup on the opposite side of the arena. Time spent sniffing each cup and time in each side chamber was recorded for each phase in seconds by Noldus Ethovision (Version 15) software.

### 2.10 Weighted door social motivation task

Social motivation was measured via the weighted door task which was adapted from Borland et al. (2017). The weighted door arena was constructed in-house by creating a 40 cm by 15 cm box (external measurements; see Supplementary Figure S1A for additional measurements) out of matte, white acrylic panels (1/4” White Opaque P95 Matte Acrylic Sheets; Canal Plastics Center) for the floor and walls. The arena was divided into two zones, a starting zone and a social zone, by a single wall with a one-way swinging door measuring 8 cm wide by 5 cm high. The dividing wall was constructed from the same white acrylic as the exterior walls. The swinging door was constructed from clear plexiglass with a plastic hinge at the top. A plexiglass, square container was attached to the bottom of the door on the social zone side so that it could be loaded with weights (0.25″ x 0.25″, 1/6 oz. tungsten cubes; Aneco). Small holes were drilled in the door to allow for olfactory detection of the social stimulus. During the trials, a novel conspecific, a stimulus mouse strain-, age-, sex-, and weight-matched to the subject mouse, was placed within an inverted pencil cup close to the back wall of the social zone so that the door was not impeded. Prior to initiating the trials, subject mice were trained to push through an unweighted door. The door was propped open slightly by placing two of the tungsten weights, the same as those used to weight the door in subsequent trials, between each corner of the door and the edge of the barrier separating the two zones (see Supplementary Figures S1B,C) which propped the door open by ¼” at the bottom edge of the door. Each mouse was individually placed in the starting zone and allowed to freely explore the arena without a time limit. Training concluded when each mouse successfully entered the door by pushing through once with the door propped open and twice without propping open the door.

Following training, each subject mouse performed 5 consecutive trials each a maximum of 3 minutes. The trial concluded when the mouse entered the social zone. At the conclusion of each trial, the mouse was removed from the arena, the arena was cleaned with 70% ethanol, and the stimulus mouse was changed to a new matched conspecific. Aside from the time spent cleaning the arena and changing the stimulus, experimental mice performed the trials consecutively without breaks. During the second trial, weight was added to the door by adding 1/3 oz. (~9.45 g) weight to the container on the door. During each of trials 3–5, an additional 1/3 oz. (~9.45 g) weight was added to the door to increase the difficulty and required effort in each trial. In all trials, weights were arranged in a single layer such that only ¼” of the transparent door was visually occluded. Latency to pass through the door and time spent within approximately 2 cm proximity to the door, indicating attempts to push the door open or investigation of the door, were recorded in seconds by Noldus Ethovision (Version 15) software.

### 2.11 Ladder task

The ladder task was used to measure social motivation and was developed in-house by constructing a 60 cm by 30 cm box (external measurements; see Supplementary Figure S2A for additional measurements) from matte, white acrylic panels (1/4” White Opaque P95 Matte Acrylic Sheets; Canal Plastics Center) for the walls and floor. Within the box, an acrylic platform attached to the top of a metal rung ladder was constructed (see Supplementary Figure S2B). The platform was able to be positioned on the floor of the box or to be positioned at varying heights which increased the steepness of the ladder (see Supplementary Figures S2C,D). During the trials, a novel conspecific, a stimulus mouse strain-, age-, sex-, and weight-matched to the subject mouse, was placed within an inverted pencil cup in the middle of the platform. Each subject mouse completed five consecutive 3-min trials. During each trial, the steepness of the ladder was increased by elevating the platform (and the top of the ladder) by 6.75 cm in each trial (from 0 cm in trial 1 to 27 cm in trial 5). Each trial ended when the subject mouse climbed the ladder and reached the platform or at the end of 3 min. At the conclusion of each trial, the mouse was removed from the arena, the arena was cleaned with 70% ethanol, and the stimulus mouse was changed to a new matched conspecific. Aside from the time spent cleaning the arena and changing the stimulus, experimental mice performed the trials consecutively without breaks. To determine whether mice were motivated by the social interaction or by the exploration of the ladder, the task was repeated 24 h later with an empty pencil cup. Social and nonsocial (empty pencil cup) conditions were compared within and across strains. Latency to reach the platform was recorded across all trials by hand in seconds.

### 2.12 Statistical analysis

Results of health, home cage behavior, and reflexes assessments are presented as percentages per strain. All other results are presented as the mean ± standard error of the mean. Data for weight, olfaction, and performance on the EPM were analyzed using two-way analyses of variance (ANOVAs), with sex and strain as the between-subjects factors. Data resulting from behavioral tests that required multiple phases or trials, including the rotarod test, three-chamber task, weighted door task, and ladder task, were analyzed using a repeated measures ANOVA (rmANOVA) with sex and strain as the between-subjects factors and phase or trial as the within-subjects factor. Data from the FDSI test were analyzed using a multivariate ANOVA (MANOVA). *Post hoc* tests were performed for each analysis using Bonferroni correction for all pairwise comparisons. Statistical analyses were performed using IBM SPSS Statistics (Version 29) software package. An alpha value of *p* < 0.05 was considered statistically significant.

## 3 Results

### 3.1 Home cage, reflex, and sensory observations

**Figure 1:**
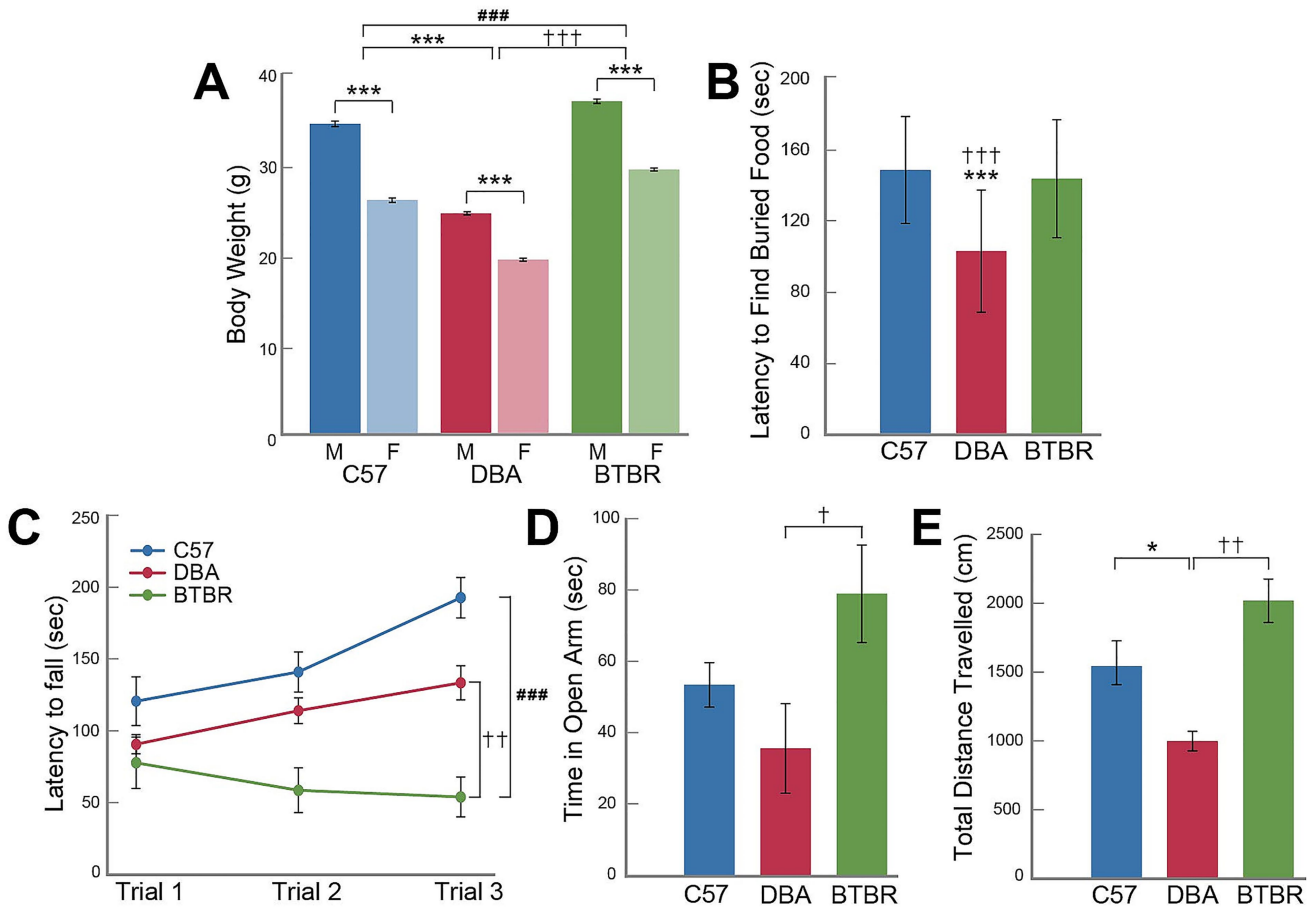
Strain differences in weight, olfaction, motor coordination, anxiety-like behavior, and hyperactivity. Data shown are the means (± SEM) for body weight prior to initiating behavioral tasks **(A)**, a fifteen-minute olfactory test to find buried food **(B)**, three five-minute rotarod trials **(C)**, and a five-minute elevated plus maze (EPM) task **(D,E)**. *N* = 20 for each strain with even number of males and females per group. Hashes indicate significant differences between C57 and BTBR mouse strains; daggers indicate significant differences between DBA and BTBR mouse strains; asterisks indicate significant differences between C57 and DBA mouse strains and differences within each strain. One, two, or three symbols (hash, dagger, or asterisk) indicate significant differences between groups at an alpha level of *p* < 0.05, *p* < 0.01, or *p* < 0.001, respectively, as indicated by pairwise *post hoc* analyses with Bonferroni correction.

All mice were observed for typical home cage behaviors, reflexes, and sensory ability to detect any potential confounding variables prior to behavioral testing. As behavioral tasks included pushing open a weighted door, the weight of each mouse across strains and sexes was compared to determine whether weight was associated with performance on these tasks. Figure 1A shows that there were significant differences in weight between sexes and strains. As expected, sex was a significant factor influencing differences in body weight (F*_1,54_* = 1451.03, *p* < 0.001). Female mice weighed less than male mice across all strains (*p* < 0.05). However, strain was also a significant factor affecting body weight (F*_2,54_* = 1318.17, *p* < 0.001). BTBR mice weighed the most followed by C57 mice and then DBA mice (*p* < 0.05, Figure 1A). Nest building also differed across strains (Pearson *χ*^2^ = 7.78, *p =* 0.02) with BTBR mice more frequently failing to shred cotton nestlets into beds (Table 1).

**Table 1:** Measurements of health, home cage behavior, reflexes, olfaction, and motor coordination suggest baseline differences between strains in weight, olfaction, and motor coordination.

Measurement	C57BL/6J	DBA/2J	BTBR
N	20	20	20
Male: Female Ratio	50:50	50:50	50:50
Avg Weight (g) Males	34.8 ± 0.84	25.01 ± 0.17	37.3 ± 0.24
Avg Weight (g) Females	26.5 ± 0.25	19.95 ± 0.17	29.84 ± 0.16
Poor coat condition	0%	0%	0%
Home cage behavior			
(% of cages exhibiting normal behavior)			
Huddling	100%	100%	100%
Nest building	95%	100%	75%*
Reflexes			
(% of mice with normal responses)			
Vibrissae Orienting Reflex	100%	100%	100%
Grasping Reflex	100%	100%	100%
Visual Placing Reflex	100%	100%	100%
Acoustic Startle Reflex	100%	100%	100%
Olfaction Tests			
(% of mice with normal responses; latency in sec)			
Uncovered Buried Food	100%	100%	100%
Latency to Find Buried Food	148.65	102.85*	143.8
Motor coordination			
(latency in sec, all trials combined)			
Rotarod Latency	151.4 ± 9.4*	112.8 ± 5.8*	63.5 ± 9.1*

Data shown are means (± SEM). Asterisks indicate significant differences (*p* < 0.05) between strains (*n* = 20 for each strain with even number of males and females per group) as indicated by pairwise *post hoc* analyses with Bonferroni correction. Rows with single asterisks indicate one strain differing from the other two while rows with three asterisks indicate significant differences between all three strains.

To examine whether olfaction differed between strains or sexes, the buried food test was performed prior to social behavior tests. As shown in Figure 1B, the latency to find buried food differed between strains (F*_2,54_* = 14.97, *p* < 0.001) such that DBA mice uncovered the novel food faster than C57 and BTBR mice (*p* < 0.001). However, no significant differences were found between C57 and BTBR mice (*p* > 0.05).

To determine any other observable differences in health or home cage behaviors, mice were observed for coat health, nest building, huddled sleeping, or grasping reflex prior to social behavior testing. No differences were observed across strains for any of these variables (see Table 1).

### 3.2 Rotarod test performance

The rotarod test was used to determine any potential differences in motor control across strains. The latency to fall from the rotarod was recorded for each mouse and averaged across three successive trials. Figure 1C shows that, while no differences were found between sexes (F*_1,54_* = 1.08, *p* = 0.30), an rmANOVA indicated a significant main effect for strain (F*_2,54_* = 17.38, *p* < 0.001). BTBR mice had lower latency to fall from the rotarod than the DBA and C57 mice (*p* < 0.001 and *p* < 0.01, respectively) suggesting lower motor performance. These results agree with published literature identifying a variety of motor impairments including deficits in fine motor skill and dystonia-like movements. Of note, previous studies using the rotarod test with BTBR mice noted observed inattentiveness during the task (Xiao et al., 2020) which may contribute to this model’s poor performance during the motor task.

### 3.3 Elevated plus maze

The elevated plus maze was used to determine any potential differences in anxiety-like behavior between strains. Figure 1D shows that there were significant differences between strains (F*_2,54_* = 3.73, *p* = 0.03) but not sexes (F*_1,54_* = 0.13, *p* = 0.72) for time spent in the open arms of the maze as indicated by a 2×3 ANOVA. *Post hoc* analysis indicated that DBA and BTBR mice significantly differed in amount of time spent in the open arms such that DBA mice spent the least time in the open arms and BTBR mice spent the most time in the open arms (*p* = 0.026). Likewise, as shown in Figures 1E, a separate 2×3 ANOVA indicated significant differences between strains (F*_2,54_* = 13.81, *p* < 0.001) but not sexes (F*_1,54_* = 0.51, *p* = 0.48) for total distance travelled in the EPM. DBA mice travelled less distance during the EPM than C57 (*p* = 0.015) and BTBR mice (*p* < 0.001).

### 3.4 Free dyadic social interaction task

**Figure 2:**
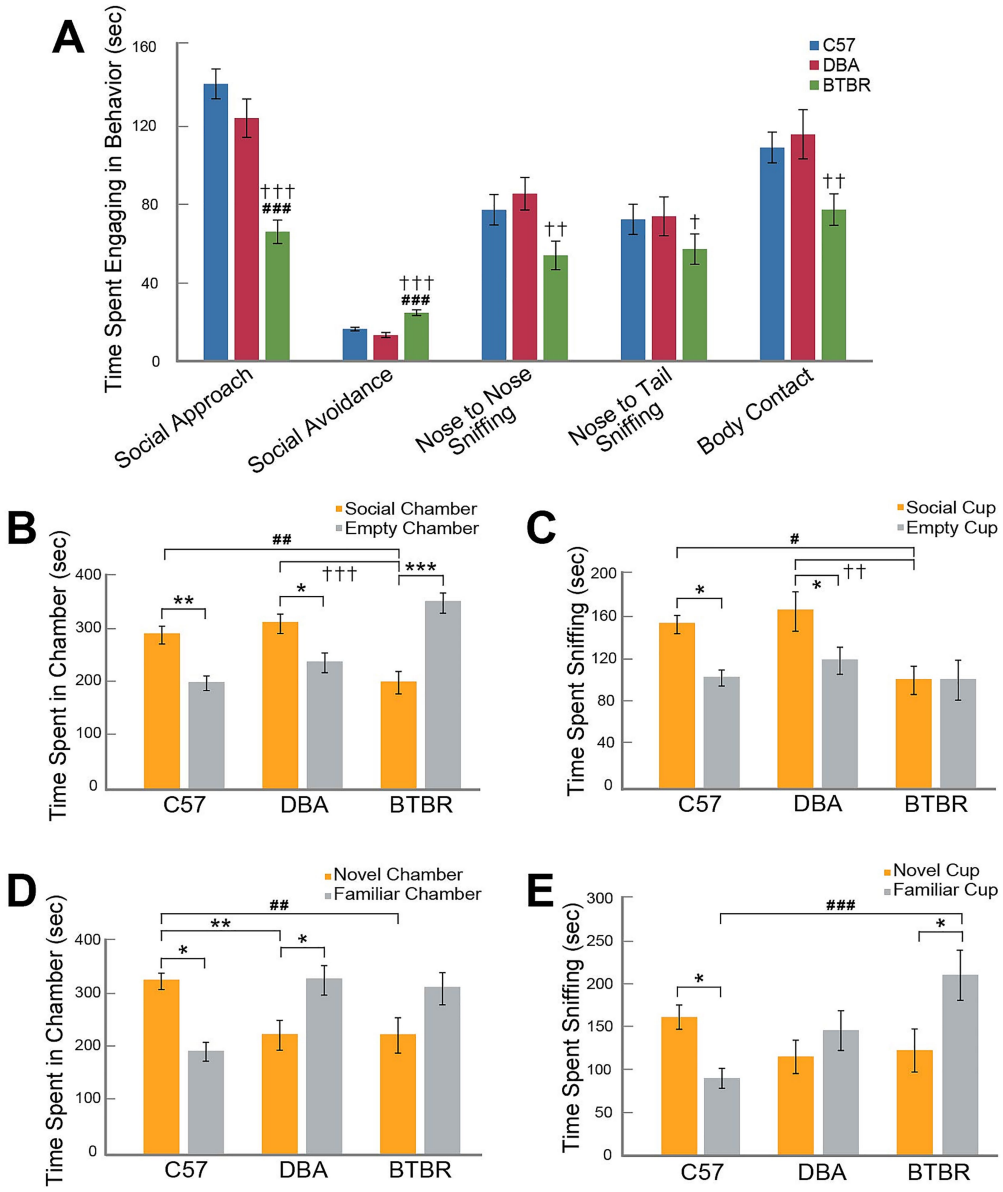
Strain differences in sociability by FDSI and three-chamber tasks. Data shown are the means (± SEM) at the conclusion of a five-minute free dyadic social interaction test (FDSI) **(A)**, a ten-minute sociability phase of the three-chamber task **(B,C)**, and a ten-minute preference for social novelty phase of the three-chamber task **(D,E)**. Number of subjects per strain for FDSI **(A)** are as follows: C57, *N* = 16 (6 males, 10 females); DBA, *N* = 19 (9 males, 10 females); BTBR, *N* = 20 (10 males, 10 females). Number of subjects per strain for the three-chamber task **(B–E)** are as follows: C57, *N* = 19 (9 males, 10 females); DBA, *N* = 20 (10 males, 10 females); BTBR, *N* = 20 (10 males, 10 females). Hashes indicate significant differences between C57 and BTBR mouse strains; daggers indicate significant differences between DBA and BTBR mouse strains; asterisks indicate significant differences between C57 and DBA mouse strains and differences within each strain. One, two, or three symbols (hash, dagger, or asterisk) indicate significant differences between groups at an alpha level of *p* < 0.05, *p* < 0.01, or *p* < 0.001, respectively, as indicated by pairwise *post hoc* analyses with Bonferroni correction.

The FDSI task was used to examine strain differences in social approach and social avoidance behaviors. A MANOVA indicated that during the FDSI task, the three mouse strains differed significantly on duration of nose-to-nose sniffing (F*_2,49_* = 4.42, *p* = 0.017), duration of anogenital sniffing (F*_2,49_* = 3.23, *p* = 0.048), duration of body contact (F*_2,49_* = 4.40, *p* = 0.017), approach (F*_2,49_* = 20.32, *p* < 0.001), and avoidance (F*_2,49_* = 26.56, *p* < 0.001). No sex differences were found on any of these behaviors. *Post hoc* analyses indicated that BTBR mice displayed lower duration times for approach than C57 and DBA mice (*p* < 0.001) and higher duration times for avoidance than C57 and DBA mice (*p* < 0.001). BTBR mice also spent less time in body contact with the social stimulus, less time nose-to-nose sniffing, and less time nose-to-tail sniffing than DBA mice (*p* = 0.006, 0.005, and 0.015, respectively; Figure 2A).

### 3.5 Three-chamber task

The three-chamber task was used to examine sociability differences between strains. During the second phase of the task measuring general sociability, significant strain (F*_2,53_* = 4.26, *p* = 0.019) but not sex (F*_1,53_* = 0.29, *p* = 0.59) differences in time spent across the social and non-social chambers, as indicated by a rmANOVA, are shown in Figures 2B,C. *Post hoc* analysis indicated that each strain significantly differed in time spent in the social and non-social chambers with C57 (*p* = 0.008) and DBA mice (*p* = 0.029) spending more time in the social chamber and BTBR (*p* < 0.001) spending more time in the nonsocial chamber. C57 and DBA mice did not significantly differ in time spent in the social chamber. However, BTBR mice spent significantly less time in the social chamber than either C57 or DBA mice (*p* = 0.002, *p* < 0.001, respectively). A separate rmANOVA indicated significant differences between strains (F*_2,53_* = 6.50, *p* = 0.003) but not sex (F*_1,53_* = 1.50, *p* = 0.23) were also found for time spent investigating the social and nonsocial pencil cups during phase 2. BTBR mice spent significantly less time investigating the social pencil cup than C57 (*p* = 0.01) or DBA mice (*p* = 0.002). No differences were found on social pencil cup investigation between C57 and DBA mice, and no differences were found between any strains for time spent investigating the empty pencil cup (*p* > 0.05; Figures 2B,C).

During the third phase of the task which measures preference for social novelty, significant strain (F*_2,54_* = 3.97, *p* = 0.025) but not sex (F*_1,54_* = 2.65, *p* = 0.11) differences were found in time spent in the familiar and novel social stimulus chambers. C57 mice spent more time in the novel social stimulus chamber (*p* = 0.013), DBA mice spent more time in the familiar social stimulus chamber (*p* = 0.048), and BTBR mice did not significantly differ in time spent between chambers. DBA and BTBR mice did not significantly differ in time spent in either the familiar or novel chamber while C57 mice differed from both DBA and BTBR mice in time spent in the familiar chamber (*p* < 0.001; *p* = 0.002, respectively) and novel chamber (*p* = 0.009; *p* = 0.009, respectively; Figure 2D). A rmANOVA indicated that strain (F*_2,54_* = 3.62, *p* = 0.034) was also a significant factor in time spent investigating the two social stimuli, but not sex (F*_1,54_* = 2.23, *p* = 0.14). C57 mice spent more time investigating the novel social stimulus as compared to the familiar social stimulus (*p* = 0.046), DBA mice did not differ in investigation of the novel and familiar social stimuli (*p* = 0.315), and BTBR mice spent significantly more time investigating the familiar social stimulus as compared to the novel social stimulus (*p* = 0.014). *Post hoc* analyses comparing differences across strains indicated that BTBR mice spent significantly more time investigating the familiar cup than C57 mice (*p* < 0.001; Figure 2E).

### 3.6 Weighted door task

**Figure 3:**
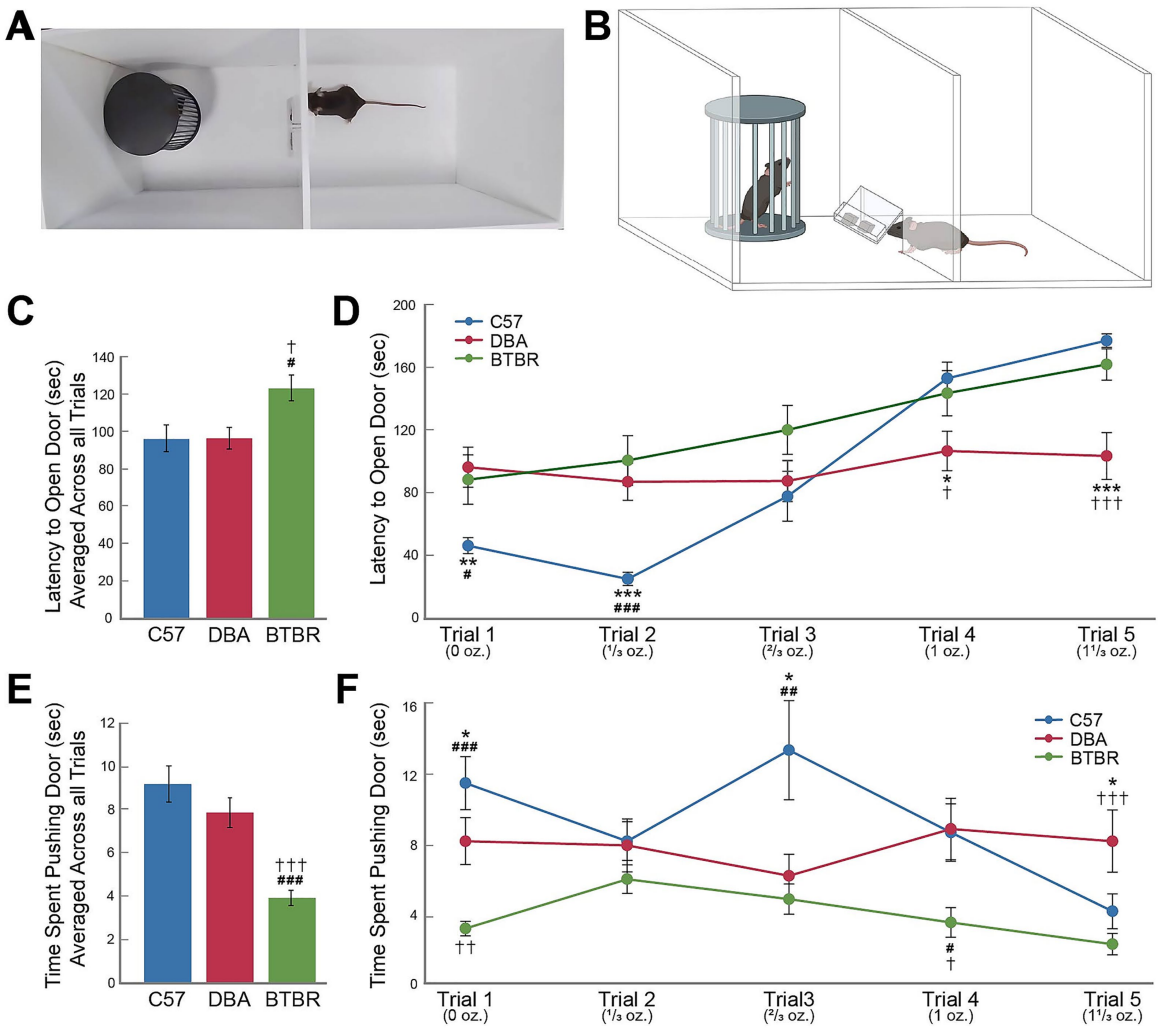
Strain differences in social motivation as measured by the weighted door task. The novel weighted door task is shown via superior view recorded by a camera **(A)** and by graphic depiction (**B**, created with BioRender.com). Data shown are the means (± SEM) at the conclusion of five three-minute weighted door trials **(C,D–F)**. *N* = 20 mice per strain with an equal number of males and females in each group. Hashes indicate significant differences between C57 and BTBR mouse strains; daggers indicate significant differences between DBA and BTBR mouse strains; asterisks indicate significant differences between C57 and DBA mouse strains. One, two, or three symbols (hash, dagger, or asterisk) indicate significant differences between groups at an alpha level of *p* < 0.05, *p* < 0.01, or *p* < 0.001, respectively, as indicated by pairwise *post hoc* analyses with Bonferroni correction.

The weighted door task was used to measure social motivation differences between the strains (see Figures 3A,B). Following the weighted door task, a rmANOVA indicated no main effect for sex (F*_1,54_* = 1.27, *p* = 0.27) and a trend towards significance for the main effect of strain (F*_2,54_* = 2.87, *p* = 0.065) on latency to enter the social chamber through the weighted door. However, *post hoc* comparisons indicated that the latency of the BTBR mice to enter the social chamber was higher compared to C57 (*p* = 0.042) and DBA mice (*p* = 0.044; Figure 3C). Pairwise comparisons examining within trial latencies across strains indicated that strain differences varied per trial. C57 mice displayed lower latency times as compared to DBA and BTBR mice on trials 1 and 2 (Trial 1, DBA: *p* = 0.006; Trial 1, BTBR: *p* = 0.02; Trial 2, DBA: *p* < 0.001; Trial 2, BTBR: *p* < 0.001). By trials 4 and 5, in which the weight of the door was highest, C57 mice demonstrated higher latency times than for all previous trials within that strain (*p* < 0.001 for all comparisons). DBA mice displayed significantly lower latencies compared to C57 and BTBR mice in trials 4 and 5 (Trial 4, C57: *p* = 0.01; Trial 4, BTBR: *p* = 0.04; Trial 5, C57: *p* < 0.001; Trial 5, BTBR: *p* < 0.001), and DBA mice did not show significant differences in latencies across the five trials within the strain (*p* > 0.05). BTBR mice demonstrated a gradual increase in latency times as the weight of the door increased such that latency times in trial 4 were significantly higher than trials 1 and 2 (*p* < 0.001 and *p* = 0.003, respectively), and latency times in trial 5 were significantly higher than trials 1–3 (Trials 1–2, *p* < 0.001, Trial 3, *p* = 0.007; Figure 3D).

A separate rmANOVA indicated significant differences for strain (F*_2,54_* = 12.09, *p* < 0.001) but not sex (F*_1,54_* = 0.03, *p* = 0.98) on time spent proximal to the weighted door. Figure 3E shows that BTBR mice spent significantly less time proximal to the weighted door than C57 (*p* < 0.001) and DBA mice (*p* < 0.001). When comparing by trial the time spent proximal to the weighted door, we found that all three groups significantly differed during trial 1 with C57 mice displaying the most time proximal to the weighted door and BTBR mice displaying the least time proximal to the weighted door (C57 compared to DBA, *p* = 0.045; C57 compared to BTBR, p < 0.001; DBA compared to BTBR, p = 0.003). In trial 3, C57 displayed significantly more time proximal to the weighted door compared to DBA and BTBR mice (p = 0.01, *p* = 0.002, respectively). In trial 4, BTBR mice displayed significantly less time near the weighted door compared to C57 and DBA mice (*p* = 0.015, *p* = 0.011, respectively). In trial 5, DBA mice spent significantly more time proximal to the weighted door compared to C57 and BTBR mice (p = 0.015, *p* < 0.001, respectively). Comparisons within strains indicated that C57 mice spent significantly less time proximal to the weighted door in trial 5 than all previous trials (*p* ≤ 0.01 for all comparisons) while DBA mice displayed no significant differences in time spent proximal to the weighted door across the five trials (*p* > 0.05 for all comparisons). BTBR mice demonstrated differences in time spent proximal to the weighted door only between trials 2 and 5 with less time spent proximal to the door in trial 5 (*p* = 0.02; Figure 3F).

### 3.7 Ladder task

**Figure 4:**
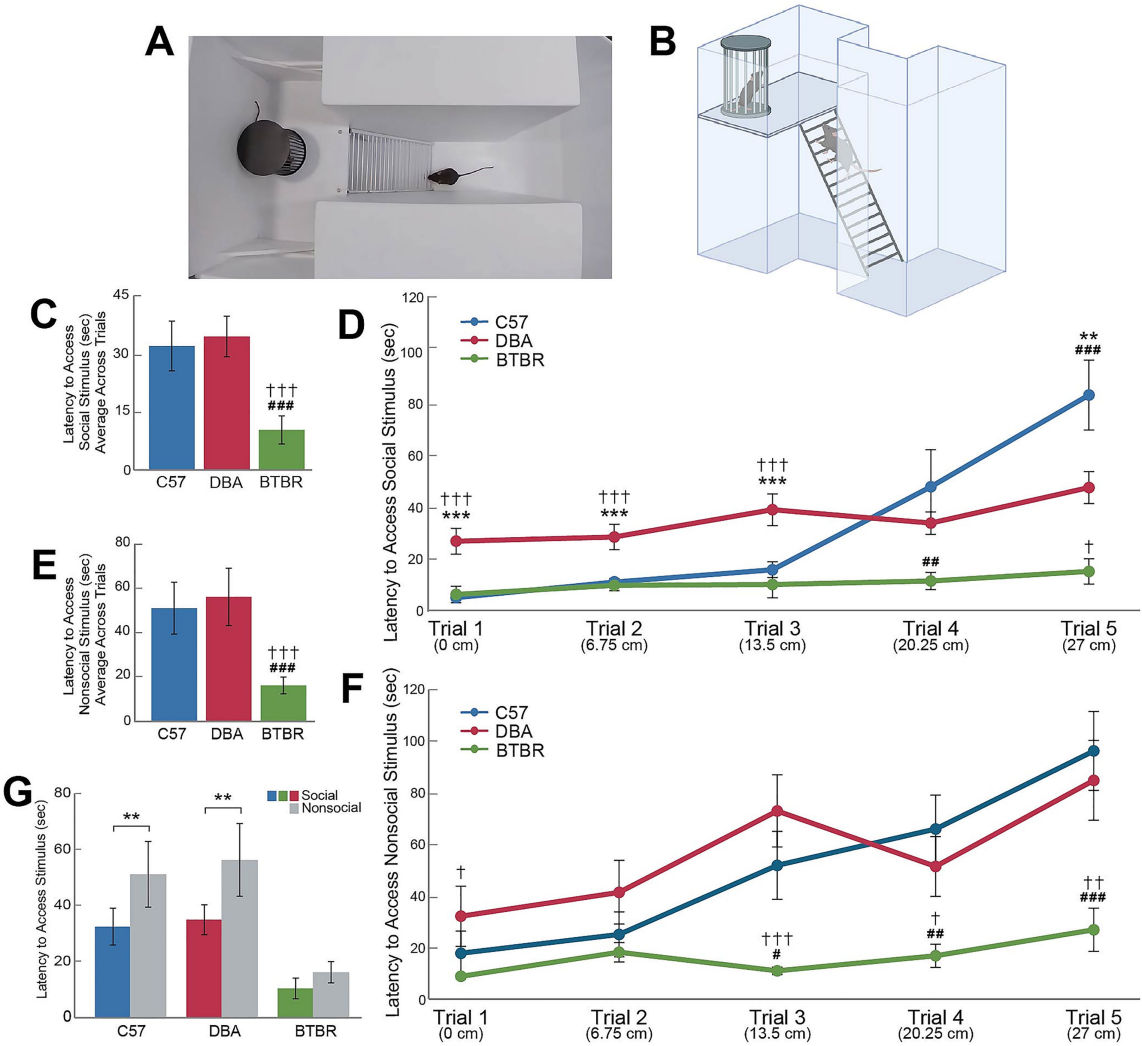
Strain differences in social motivation as measured by the ladder task. The novel ladder task is shown via superior view recorded by a camera **(A)** and by graphic depiction (**B**, created with BioRender.com). Data shown are the means (± SEM) at the conclusion of each of the five three-minute ladder task trials in two conditions: a social condition with a social stimulus at the top of the ladder and a nonsocial condition with an empty pencil cup at the top of the ladder **(C,D–G)**. *N* = 20 mice per strain with an equal number of males and females in each group. Hashes indicate significant differences between C57 and BTBR mouse strains; daggers indicate significant differences between DBA and BTBR mouse strains; asterisks indicate significant differences between C57 and DBA mouse strains and differences within each strain. One, two, or three symbols (hash, dagger, or asterisk) indicate significant differences between groups at an alpha level of *p* < 0.05, *p* < 0.01, or *p* < 0.001, respectively, as indicated by pairwise *post hoc* analyses with Bonferroni correction.

The ladder task was used to examine differences in social motivation between strains (see Figures 4A,B). An rmANOVA indicated significant strain (F*_2,54_* = 17.21, *p* < 0.001) but not sex effects (F*_1,54_* = 0.85, *p* = 0.77) for latency to climb the ladder over the five trials using social and nonsocial stimuli at the top of the ladder. The within-subjects factor of stimulus type (social or nonsocial) was also significant (F*_1,54_* = 16.40, *p* < 0.001, with Greenhouse–Geisser correction). Figure 4C shows that during the social trials, BTBR mice displayed lower latency times to reach the top of the ladder than C57 (*p* < 0.001) and DBA mice (*p* < 0.001). When comparing latency to climb the ladder across each strain within each social trial, DBA mice displayed significantly higher latencies during trials 1, 2, and 3 as compared to C57 and BTBR mice (*p* < 0.001 for all comparisons). In the social condition trial 4, BTBR mice displayed lower latencies as compared to C57 mice (*p* = 0.005). In the social condition trial 5, all strains significantly differed in latency to climb the ladder (C57 compared to DBA, *p* = 0.006; C57 compared to BTBR, *p* < 0.001; DBA compared to BTBR, *p* = 0.012; Figure 4D). Comparing within strains, BTBR mice did not show significant differences in latency to reach the social stimulus across trials (*p* > 0.05). DBA mice displayed a moderate increase in latency to reach the social stimulus across trials such that latencies in trial 5 (the steepest trial) were significantly higher than all other trials except trial 3 (*p* = 0.03 for all comparisons). C57 mice, however, displayed more marked differences as trial difficulty increased with significantly higher latencies to reach the social stimulus in trials 4 and 5 as compared to trials 1, 2, and 3 (*p* ≤ 0.001 for all comparisons).

Figure 4E shows that during the nonsocial trials, BTBR mice displayed lower latency times to reach the top of the ladder as compared to C57 (*p* < 0.001) and DBA mice (*p* < 0.001). When comparing the latency to climb the ladder across each strain within each nonsocial trial, latencies to reach the top of the ladder did not differ between C57 and DBA mice in any trial (*p* > 0.05 for all comparisons). In nonsocial trial 1, BTBR mice displayed lower latencies as compared to DBA mice (*p* = 0.049). BTBR mice displayed lower latencies in nonsocial trials 3, 4, and 5 as compared to C57 and DBA mice (Trial 3, C57: *p* = 0.01; Trial 3, DBA: *p* < 0.001; Trial 4, C57: *p* = 0.001; Trial 4, DBA: *p* = 0.02; Trial 5, C57: *p* < 0.001; Trial 5, DBA: *p* = 0.003; Figure 4F). Figure 4G shows that both C57 and DBA mice displayed significant differences in social and nonsocial latencies across all trials (*p* = 0.006 and *p* = 0.002, respectively), but BTBR mice did not display significant differences in social and nonsocial latencies (*p* = 0.38).

## 4 Discussion

Given that reduced or irregular social interactions are found in multiple neuropsychiatric and neurodevelopmental conditions, the importance of increasing our ability to quantify specific social behaviors in mouse models of these conditions is increasingly clear. The current study proposes the use of the weighted door task and ladder task for the measurement of social motivation in mice and to complement established behavioral measures of social reward and social cognition. The findings of this study provide social motivation phenotypes for three common inbred strains: C57, DBA, and BTBR.

### 4.1 Assessment of non-social behavior

As both proposed social motivation measures involve physical manipulation, motor coordination, and sensory function, preliminary observations of strain differences in general health, motor control, and olfaction were conducted to identify any confounding variables that might affect their performance on these tasks. While the DBA mice weighed less at the initiation of the behavioral tasks, this strain had the lowest latency times to open the door at the heaviest weights suggesting that the difference in body weight did not impair their performance. Likewise, no sex differences were found in the weighted door task suggesting that the lower weight of the female mice in each strain did not impact their ability to complete the weighted door task.

When testing olfactory function, C57 and BTBR mice displayed no significant differences in the latency to find buried food, while DBA mice displayed decreased latency to find buried food compared to C57 and BTBR mice. This finding agrees with previous research showing that DBA mice uncover buried food faster than C57 mice (Moy et al., 2007). As C57 mice are not described in previous research as commonly having impaired olfactory function, and as the C57 mice were able to find the buried food in every case, the significant difference between C57 and DBA latency times was interpreted as increased sensitivity for DBA mice and not decreased sensitivity for C57 mice (Machado et al., 2018; Moy et al., 2004, 2007). Also, as BTBR mice displayed no significant differences in latency to find buried food, as compared to C57 mice, and found the buried food in every case, BTBR mice were interpreted to have typical olfactory function. This finding also agrees with previous working demonstrating that BTBR mice can distinguish different olfactory stimuli (Moy et al., 2008a,b). Together, these findings suggest that olfactory function was not impaired in any of the strains and therefore not a confounding factor that would impede the use of olfactory social cues during social behavior measurement.

When assessing motor coordination, BTBR mice had a significantly lower latency to fall off the rotarod compared to the other strains suggesting a deficit in motor ability which agrees with previous research studies demonstrating motor deficits in BTBR mice as measured by the rotarod task (Xiao et al., 2020). It is possible that these motor deficits are partially dependent on motor learning deficits. Kiffmeyer et al. (2022) demonstrated that BTBR mice express differences in cerebellar morphology as compared to C57 mice and that BTBR mice demonstrated impaired cerebellum-dependent motor learning. BTBR mice have also been shown to have impaired attention and learning in other tasks such as object-based attention tasks (Chao et al., 2018; McTighe et al., 2013). It is notable that during the rotarod test conducted in the current study, BTBR mice initially did not differ in latency to fall from DBA mice during trial 1; however, both the DBA and the C57 mice improved their performance across the three trials while the BTBR mice did not. However, despite potential confounds of motor control and motor learning deficits, BTBR mice had the lowest latencies across the three strains in reaching the top of the ladder task in both social and nonsocial conditions, and the latency for BTBR mice to open the weighted door during the lowest weight trials did not significantly differ from DBA mice. Likewise, nearly 100% of the BTBR mice completed the ladder task in both the social and nonsocial conditions (Supplementary Table S1). Together, these observations suggest that while motor coordination was a potential confounding variable, BTBR mice were able to complete the weighted door and ladder tasks as efficiently as the two other strains.

In the EPM, DBA mice spent the least amount of time in the open arms suggesting increased anxiety-like behavior. Elevated anxiety-like behavior may have influenced some social behavior measurements in this strain. For example, despite preferring a social chamber over a nonsocial chamber in the sociability phase of the three-chamber task, DBA mice failed to display a preference for a novel social stimulus over a familiar one. Additionally, DBA mice displayed longer latencies in the initial trials of both the weighted door task and the ladder task during the social condition as compared to C57 mice. This observation could partially be explained by higher anxiety-like behaviors in the DBA strain inducing a hesitancy to initiate a new task. Conversely, BTBR mice did not display anxiety-like behavior in the EPM task as this strain spent the most time in the open arms. BTBR mice have previously been described as having variable behavior outcomes during anxiety measures ranging from anxiogenic to anxiolytic with some reporting increased stress-reactivity (Benno et al., 2009; Chao et al., 2018; McFarlane et al., 2008; Moy et al., 2007; Pobbe et al., 2011; Silverman et al., 2010; Yang et al., 2009; Yang et al., 2012). Our data also indicated that the BTBR mice were consistently willing to complete the ladder task with very low latency times compared to C57 and DBA mice, even in early trials when the ladder task arena was a novel environment. While this behavior could be explained by reduced anxiety-like behavior, it is also potentially confounded by general hyperactivity. The total distance traveled in the EPM was higher for the BTBR mice and previous studies have shown that BTBR mice display hyperactivity (Khan et al., 2025). Together, these observations suggest that anxiety was likely not a confounding variable for BTBR mice during the weighted door and ladder tasks, but potential confounding effects of stress-reactivity and locomotor hyperactivity remain a possibility for this strain.

### 4.2 Assessment of social interaction

The FDSI and the three-chamber tasks were used to evaluate classic social behaviors including social approach, social avoidance, sociability, and preference for social novelty. In the FDSI task, BTBR mice displayed fewer social approach behaviors, more social avoidance behaviors, less social sniffing, and reduced body contact time as compared to C57 and DBA mice. In the three-chamber task, BTBR mice displayed low sociability and a preference for a familiar stimulus over a novel one. These data agree with previous research demonstrating a low sociability phenotype for the BTBR strain when using the three-chamber task, social interaction tasks, and other measurements such as odor discrimination and social play (Arakawa, 2023; Bolivar et al., 2007; Khan et al., 2025; Moy et al., 2007; Pobbe et al., 2010; Yang et al., 2012). While, C57 and DBA mice did not demonstrate differences in social behavior in the FDSI task, the two strains did differ in their social behavior in the three-chamber task. Both C57 and DBA mice demonstrated a preference for the social chamber over the empty chamber, but DBA mice failed to demonstrate a preference for social novelty in the third phase of the task. While previous research findings on preference for social novelty has been mixed for DBA mice, previous research has also found a lack of preference for social novelty for this strain which has been noted to potentially be related to this strain’s increased anxiety (Moy et al., 2008a,b; Nadler et al., 2004). Thus, as measured by the FDSI and three-chamber tasks, the three inbred strains tested represent three distinct social behavior phenotypes that could then be further described by the proposed social motivation measures.

### 4.3 Assessment of social motivation

The weighted door and ladder tasks were used to measure social motivation. These tasks hold unique advantages when used in conjunction with measures of social reward and social cognition to ensure the measurement of the full breadth of social behavior phenotypes. In the current study, we found that BTBR mice displayed low social motivation. BTBR mice displayed significantly longer latencies to open the weighted door and lower time spent proximal to the weighted door task as compared to C57 and DBA mice. The BTBR mice had very low latencies to reach the top of the ladder in the ladder task, but latency times did not differ between social and nonsocial stimuli suggesting that their ladder climbing was exploratory instead of incentivized by the social partner. Together, these data expectedly align with the behavioral outcomes of BTBR mice in the standard three-chamber and FDSI social behavior tasks indicating both low social reward and low social motivation in BTBR mice. Importantly, despite previously reported learning impairments and motor control deficits, BTBR mice were able to complete the weighted door and ladder task without difficulty indicating that these behavioral measures can be used with mouse models of autism and other mouse models with similar impairments. Additionally, just as C57 and DBA mice displayed unique sociability and social novelty preferences in the three-chamber task, C57 and DBA mice displayed unique social motivation phenotypes. Both strains expectedly demonstrated higher social motivation than BTBR mice, but C57 mice displayed a dual phenotype in which less difficult trials requiring less effort prompted C57 mice to demonstrate higher social motivation. In these lower effort trials, C57 mice displayed low latencies to open the weighted door and higher times spent proximal to the weighted door task. In the ladder task, C57 mice displayed low latency times to reach the top of the ladder in the social conditions, again suggesting high social motivation. However, as trials progressed in difficulty, C57 mice displayed lower social motivation as compared to DBA mice. During trial three in the weighted door task, C57 mice increased latency times to enter the door and displayed a large increase in time spent proximal to the weighted door. This was then followed by declining time spent proximal to the weighted door and increasing latencies. Similarly in the social condition ladder task, latencies to reach the top of the ladder significantly increased following trial three. In contrast, DBA mice did not have large shifts in social motivation according to the trial difficulty. In the weighted door task, neither latencies to open the door nor time spent proximal to the weighted door significantly differed across trials for DBA mice. In the ladder task, latencies to reach a social partner did not differ across trials for DBA mice with the exception of the final and most difficult trial. Together, these findings suggest that, while DBA mice do not display a preference for social novelty in the three-chamber task, this strain has high social motivation to reach a social partner. As the behavioral outcomes of these novel behavioral tasks do not contradict standard social behavior measurements but instead demonstrate both divergent and complementary behavioral outcomes, we argue for their addition to standard social behavior measures to compose a more complete battery of social behavior tests that will measure across all three social behavior categories: social reward, social cognition, and social motivation.

### 4.4 Limitations of the study

The current study proposes important, novel behavioral measures of social motivation in mice that may allow new insights into social behavior phenotypes of mouse models. However, potential limitations of the study should also be considered in the interpretation of the current findings and in the use of these methods in future studies. First, potential confounds across strains exist such that strains with learning and attention deficits, motor coordination deficits and motor activity differences introduce confounding factors that cannot be separated from the social behavior results. In the current study, BTBR mice displayed reduced motor coordination and hyperactivity which may account for or interact with measured behavior in the weighted door and ladder task. BTBR mice are also noted to have learning and attention deficits which could suggest that the performance of this strain in the social motivation measures represents failed learning instead of decreased social motivation. More generally, while the weighted door and ladder tasks represent non-conditioned behavioral measures of social motivation, attention and learning are likely still mediating factors in the performance of these tasks. Therefore, the interpretation of these measures may be limited in certain mouse models exhibiting cognitive deficits.

Second, potential confounds exist in the procedures for weighted door and ladder task in the current study that could be limited in future studies by altered methodology. The weighted door task was not repeated with a non-social stimulus as was the ladder task. As pilot studies using the weighted door task indicate higher latency times to enter the weighted door for a nonsocial stimulus or empty pencil cup in place of a social stimulus, the findings from the current study are limited in interpretation due to the lack of this nonsocial comparison. Future studies should incorporate a nonsocial stimulus for comparison and to better expand the potential interpretation of these behavioral results. The ladder task was not video recorded in the current study, and latency times were the only outcome measure recorded. However, time spent climbing, time on the platform, or time spent sniffing the social stimulus would be potentially informative outcome measures. The non-social trials of the ladder task only included an empty pencil cup, but to account for novelty, a novel object could be used in place of a social stimulus. In the ladder task the non-social stimulus trials followed the social stimulus trials, and in both the weighted door task and the ladder task, the trials always increased in difficulty. As a potential repeated testing effect could influence a mouse’s social motivation, either increasing or decreasing motivation over time, future studies using these measurement tools may benefit from counterbalancing stimulus type and trial difficulty.

### 4.5 Conclusion

Together, these findings demonstrate the feasibility of utilizing social motivation tasks in mouse models as a complement to established sociability tasks measuring social reward and social cognition. The novel weighted door task and ladder task used in the current study were able to demonstrate the known social deficits in the BTBR inbred mouse strain while also identifying a unique and quantifiable social motivation phenotype for the C57 and DBA inbred strains. The weighted door task and ladder task should be utilized in the future to create more complete ethograms of mouse model social behavior which may then allow for a fuller understanding of the underlying neurobiology and improved pre-clinical evaluation of novel therapeutics for social symptoms in clinical populations.

## Data Availability

The raw data supporting the conclusions of this article will be made available by the authors, without undue reservation.
