# Supplementary material for: Novel behavioral tasks for the measurement of social motivation in mice: a comparison across strains
Source: Front Behav Neurosci. 2026 Jan 6;19:1678147. doi: 10.3389/fnbeh.2025.1678147 (PMC12816312; doi:10.3389/fnbeh.2025.1678147)
Supplement: Supplementary file 1 [file Data_Sheet_1.pdf]

Supplementary Material

Supplementary Figures and Tables

	C57BL/6J	DBA/2J	BTBR
Percent Completion of Weighted Door Task			
Trial 1	100%	75%	75%
Trial 2	100%	90%	65%
Trial 3	75%	80%	45%
Trial 4	30%	75%	25%
Trial 5	5%	65%	25%
Percent Completion of Ladder Task – Social Condition			
Trial 1	100%	100%	100%
Trial 2	100%	100%	100%
Trial 3	100%	100%	100%
Trial 4	85%	100%	100%
Trial 5	85%	100%	100%
Percent Completion of Ladder Task – Nonsocial Condition			
Trial 1	95%	90%	100%
Trial 2	95%	95%	100%
Trial 3	85%	95%	100%
Trial 4	85%	95%	100%
Trial 5	70%	75%	95%

Supplementary Table 1. The completion rates per strain are shown for each trial of the weighted door task and ladder task. Completion of the weighted door task was defined as opening the door within three minutes. Completion of the ladder task was defined as reaching the platform at the top of the ladder within three minutes.

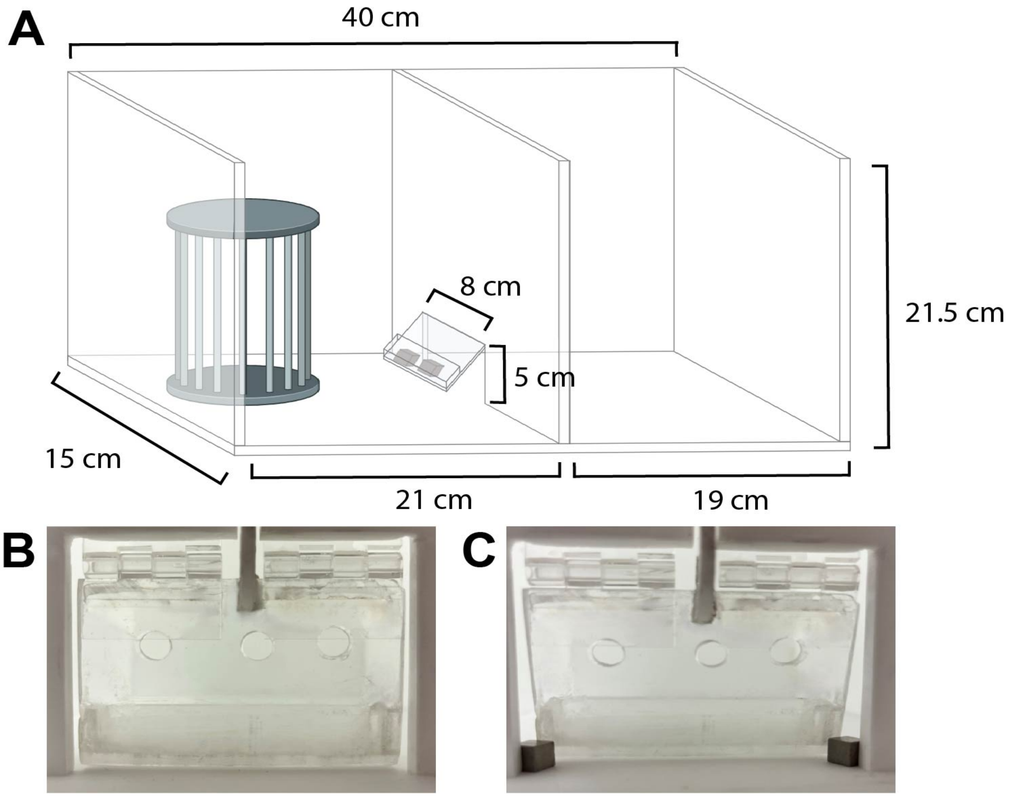

Supplementary Figure 1. The weighted door arena was constructed from matte, white acrylic panels (1/4" White Opaque P95 Matte Acrylic Sheets; Canal Plastics Center; https://www.canalplastic.com/products/7508-white-opaque-p95-acrylic-sheet?variant=32918636686) according to the dimensions depicted in Figure 1A (shown without the front-facing wall for clarity; created with BioRender.com). Figure 1B shows the one-way swinging door (shown from the viewpoint of the starting zone) constructed of clear plexiglass (1/8" Clear Colorless Acrylic Sheet, Canal Plastics Center; https://www.canalplastic.com/products/clear-colorless-p95-acrylic-sheet?variant=32772166094) with a clear plexiglass box measuring 3/8" in depth and ½" in width was constructed on the side facing the social stimulus. Three holes ¼" in diameter were drilled through the door to allow for olfaction, and the door was welded onto clear acrylic hinges (BBTO; https://www.amazon.com/dp/B087PG17GP/). The clear acrylic hinges were then welded onto the dividing wall. All welding was done by applying Weld-On 4 Acrylic Adhesive (Weld-On Adhesives Inc.; https://www.amazon.com/dp/B0096T6P1Y/) between the acrylic pieces. Tungsten cube weights (Aneco; https://www.amazon.com/dp/B083VZGHR5/) measuring 0.25" x 0.25" and weighing 1/6 oz were placed in the acrylic box on the door with the first trial including zero weights and two weights (a total of 1/3 oz) being added for each subsequent trial. In the social zone, a social stimulus was placed within an inverted pencil cup (Spectrum Diversified Store, https://www.amazon.com/dp/B00B1ZPT0Y/). To train each mouse to push open the weighted door to access the social stimulus, two of the tungsten weights were placed at each of the bottom corners of the weighted door to prop the door open, shown in Figure 1C. Once the mouse had successfully entered the door once while propped open and twice without propping, the weighted door trials were initiated.

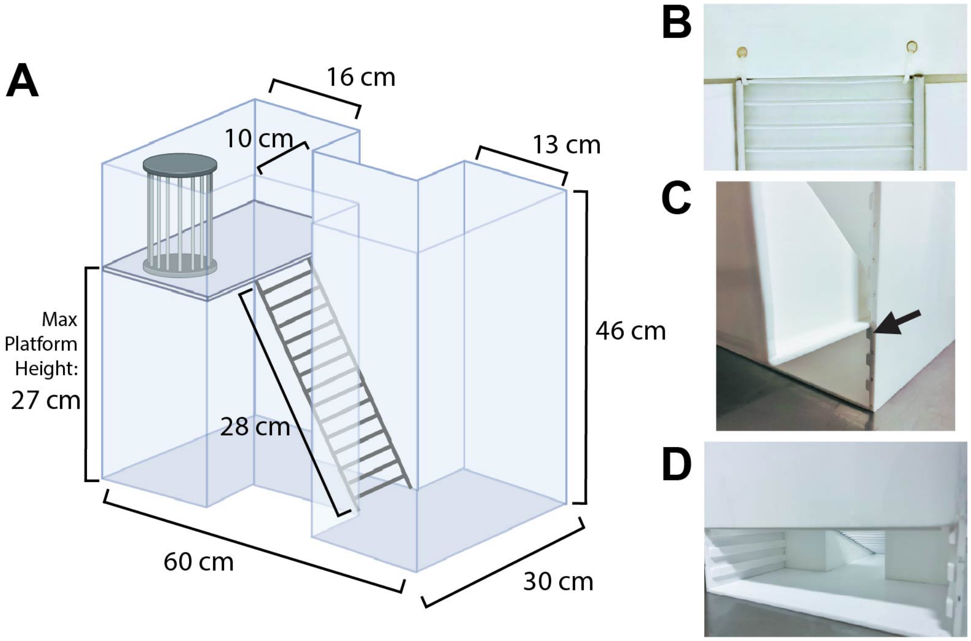

Supplementary Figure 2. The ladder task arena was constructed from matte, white acrylic panels (1/4” White Opaque P95 Matte Acrylic Sheets; Canal Plastics Center; https://www.canalplastic.com/products/7508-white-opaque-p95-acrylic-sheet?variant=32918636686) according to the dimensions depicted in Figure 1A (created with BioRender.com). Acrylic pieces were welded together by applying Weld-On 4 Acrylic Adhesive (Weld-On Adhesives Inc.; https://www.amazon.com/dp/B0096T6P1Y/) between the acrylic pieces. A metal ladder was created by utilizing metal shelving (ClosetMaid; https://www.lowes.com/pd/ClosetMaid-4-ft-x-12-in-White-Wire-Shelf/1090937) and was attached by straps onto the acrylic platform shown in Figure 2B. Brackets were constructed from the matte acrylic to support the platform at multiple heights and allowing the ladder to be held at varying degrees of steepness. Figure 2C shows the brackets, one of which is indicated by an arrow, and the platform being placed into one pair of brackets. Figure 2D shows the back side of the platform after it has been placed in a pair of brackets. Ladder heights and ladder angle from the floor of the arena for each of the five trials was as follows: Trial 1: platform elevation of 0 cm, ladder angle of 0°; Trial 2: platform elevation of 6.75 cm, ladder angle of ~14°; Trial 3: platform elevation of 13.5 cm, ladder angle of ~29°; Trial 4: platform elevation of 20.25cm, ladder angle of ~46°; Trial 5: platform elevation of 27cm, ladder angle of ~75°. A social stimulus was placed on the platform in an inverted wire pencil cup (Spectrum Diversified Store, https://www.amazon.com/dp/B00B1ZPT0Y/).
